# Chatbot Versus Lecture in the Teaching of Endodontic Diagnosis for Undergraduate Students—A Pilot Study

**DOI:** 10.1002/jdd.13940

**Published:** 2025-05-19

**Authors:** Marcelo Santos Coelho, Guilherme Bilantini Piva, Rodrigo Arruda Vasconcelos, Cássia Cestari Toia, Lucas Santos Zambon, Sigisfredo Brenelli

**Affiliations:** ^1^ Endodontic Department São Leopoldo Mandic School of Dentistry Campinas Brazil; ^2^ School of Medicine São Paulo State University Paulo Brazil; ^3^ São Leopoldo Mandic School of Medicine Campinas Brazil

**Keywords:** chatbot, diagnosis, education, endodontics, lecture, undergraduate

## Abstract

**Purposes:**

This study compared a chatbot with an expository interactive lecture as a tool for teaching pulpal and periapical diagnosis in undergraduate dental education.

**Methods:**

A chatbot and an expository interactive lecture were used to deliver the topic of pulpal and periapical diagnosis based on the American Association of Endodontics guidelines. A total of 24 second‐year students in a 4‐year undergraduate program were enrolled. An initial test (Test A) with 10 multiple‐choice questions was applied to all students. Then, the students were randomly assigned to two different groups: Lecture (control) and Chatbot (experimental). The Lecture group attended an expository interactive lecture delivered by an endodontist. Simultaneously, in the Chatbot group, the chatbot was delivered to the students through the Telegram Messenger application. After 50 min, both groups were submitted to the same test (Test B). Subsequently, the Control group used the chatbot, while the Experimental group attended a lecture by the same faculty. After the split activity, all the students replied to a questionnaire with their perceptions regarding both activities. Statistical analysis was performed with the significance level set at 5%.

**Results:**

Twenty‐two students replied to the questions. Both Lecture and Chatbot groups showed significant grade improvement (Lecture: from 6.18 ± 2.08 to 8.45 ± 1.28; Chatbot: from 5.55 ± 1.63 to 7.91 ± 1.58). No difference in the initial and final average grades was detected between the groups. Overall, the chatbot was considered more fun and simpler while the lecture was preferred for understanding (*p* < 0.05). Chatbot was rated 4.95/5 for ease of use.

**Conclusions:**

The chatbot was as effective as an interactive lecture in delivering the basic content of pulpal and periapical diagnosis. The students' perception was that the chatbot was simpler and more fun than the lecture; however, the interactive lecture is a better tool to fully understand the topic. The professor is irreplaceable when discussing the content.

## 1 Introduction

Endodontic diagnosis is paramount for treatment planning and prognosis [1]. Undergraduate students should be able to differentiate the normal pattern of pulpal and periapical tissues from the pathological features. This differentiation is achieved with the understanding of clinical signs and symptom and radiographic examination. Theoretical knowledge of endodontic content is still an area that should be given attention to [2].

Keeping undergraduate students motivated with traditional teaching is a great challenge in modern education. The time lapse to keep them concentrated seems to be too small to deliver such important content only with lectures. To circumvent this issue more attention has been given to learning styles and active methods of teaching have been introduced to undergraduate students in the past few years [3]. Chatbots are one of these emerging technologies to enhance the learning of undergraduate students [4, 5]. Created in the 1960's, chatbots are software able to emulate written or spoken conversations [6].

Recently, a Generative Pre‐trained Transformer chat (chat GPT) has proved to be superior to traditional scientific sources in the topic of oral radiation [7]. Chatbots based on a neural network model, however, present some drawbacks when delivering health content. While their reliability seems to be satisfactory, there are regional and technical aspects that can lead to a misdiagnosis. These errors could induce unexperimented students to receive inappropriate content in their curriculum. Different models of chatbots have been assessed regarding their reliability in endodontic information for the lay public [8]. While the results were satisfactory, care should be taken to avoid misinformation. Under proper supervision and with control of the content, artificial intelligence can be helpful in teaching endodontics [9, 10].

A chatbot based on specific content and a reliable source, instead of AI created, can be designed with specific questions and answers, assuring that the content is under the control of the faculty who created this chatbot. Therefore, this study aimed to compare a chatbot with an expository interactive lecture in the efficiency to deliver the topic of endodontic diagnosis among undergraduate students. Moreover, the perception of the students about both methods of teaching was assessed. The null hypothesis tested were: (1) the chatbot was equal to the lecture in enhancing the students' performance, and (2) the perception of the students was similar for the chatbot and lecture.

## 2 Materials and Methods

This study was reviewed and approved by the ethics committee of our institution (82775424.0.0000.5374). The participants involved were the 24 students of the 2nd semester of the 2nd year of an undergraduate dental program with a 4‐year duration. The whole experiment occurred in a single 4‐h session during preclinical endodontics.

**FIGURE 1:**
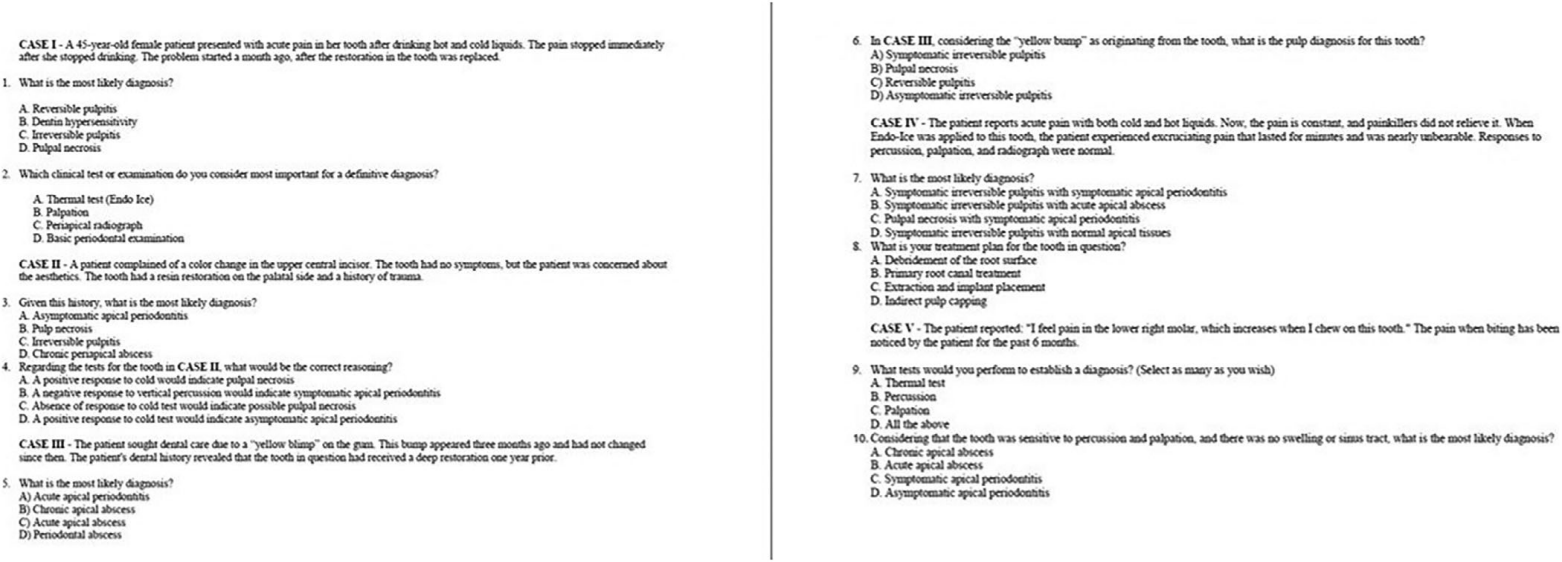
Test encompassing 10 multiple‐choice questions was applied to all students.

After signing an inform consent, an initial test (Test A) on the topic of endodontic diagnosis encompassing 10 multiple‐choice questions was applied to all students (Figure 1). This test is a simplified version of the one proposed by Alobaoid et al. [11]. All the tests had the same questions both in content and in order of the questions. The students were required to finish the test in 10 min and the tests were collected from the students. After the first test the students were randomly assigned to two different groups. A control group (Lecture group), attending an interactive lecture and an experimental group (Chatbot group) dealing with a chatbot.

The Lecture group was entitled to attend a 50‐min expository lecture with a faculty member randomly assigned among the faculty members of the endodontic department. This faculty was not involved in the conception of the research, also, he had no knowledge of the questions of the tests nor of the questionnaire. The faculty member was instructed to use the terminology and images available in the guideline of the Brazilian Endodontic Society [12]. This guideline is based on the AAE guidelines, with different images, but similar content [13]. Also, he was instructed to deliver the lecture in a dialogic fashion and to open for questions at the end of the presentation should any student still deem necessary. After the end of the presentation the students took a second test (Test B), identical to the Test A.

In the Chatbot group the students were instructed how to use the application. Basically, the chatbot was designed with a sequence of clinical examples of chief complaints Supporting Information. Following the responses of the complaints the students were led to the diagnosis of pulp diseases (reversible pulpitis, symptomatic irreversible pulpitis, asymptomatic irreversible pulpitis, or pulp necrosis). Also, clinical cases led to apical signs and symptoms of symptomatic apical periodontitis, asymptomatic apical periodontitis, acute apical abscess, or chronic apical abscess. At the conclusion of each case, a figure with a clinical and radiographic example and a written definition was displayed. Care was taken to ensure that the images used with the Chatbot group were also used in the power point presentation of Lecture group. The students were required to use the application for at most 50 min without any questions to the faculty member supervising the activity. However, technical issues could be resolved if necessary. When the students felt confident to take Test B they could make it at any moment.

After the conclusion of Test B, the students were instructed to alternate the activities. That said, the students remained in their original classrooms and the faculty member switched places. Then, the same professor delivered the lecture to the Chatbot group and the students of the Lecture group were instructed by the faculty in charge of the application on how to use the application of the chatbot. Again, at the same time, 50 min were reserved for each activity. At the end of the activity, both groups filled in a questionnaire with 12 statements about the lecture and 13 statements about the chatbot with a 5‐point Likert scale (1‐ *strongly disagree*; 2‐ *disagree*; 3‐ *neutral*; 4‐ *agree*; 5‐ *strongly agree*). Also two open‐end questions were placed questioning the drawbacks using the chatbot or lecture and the greatest advantage of the chatbot or lecture. The questionnaire applied is depicted in Tables 1 and 2.

**TABLE 1:** Questionnaire regarding the impression of the students about the activity with the chatbot.

	Strongly disagree	Disagree	Neutral	Agree	Strongly agree
Chatbot helps me to focus on the topic					
Chatbot motivates me to learn more					
Learning with Chatbot is fun					
Chatbot enhanced my understanding on the topic					
Chatbot helps me to retain knowledge					
Chatbot simplifies complex topic					
Chatbot makes the learning process easier					
Chatbot keeps me active during the learning					
Chatbot is an effective tool to provide feedback					
Chatbot is an effective tool to correct my doubts on the topic					
Chatbot is an effective tool for a reflexive learning					
Chatbot might be an effective tool, but needs improvement					
I could easily use the Chatbot					

**TABLE 2:** Questionnaire regarding the impression of the students about the lecture attended.

	Strongly disagree	Disagree	Neutral	Agree	Strongly agree
Lecture helps me to focus on the topic					
Lecture motivates me to learn more					
Learning with lecture is fun					
Lecture enhanced my understanding on the topic					
Lecture helps me to retain knowledge					
Lecture simplifies complex topic					
Lecture makes the learning process easier					
Lecture keeps me active during the learning					
Lecture is an effective tool to provide feedback					
Lecture is an effective tool to correct my doubts on the topic.					
Lecture is an effective tool for a reflexive learning					
Lecture might be an effective tool, but needs improvement					

**FIGURE 2:**
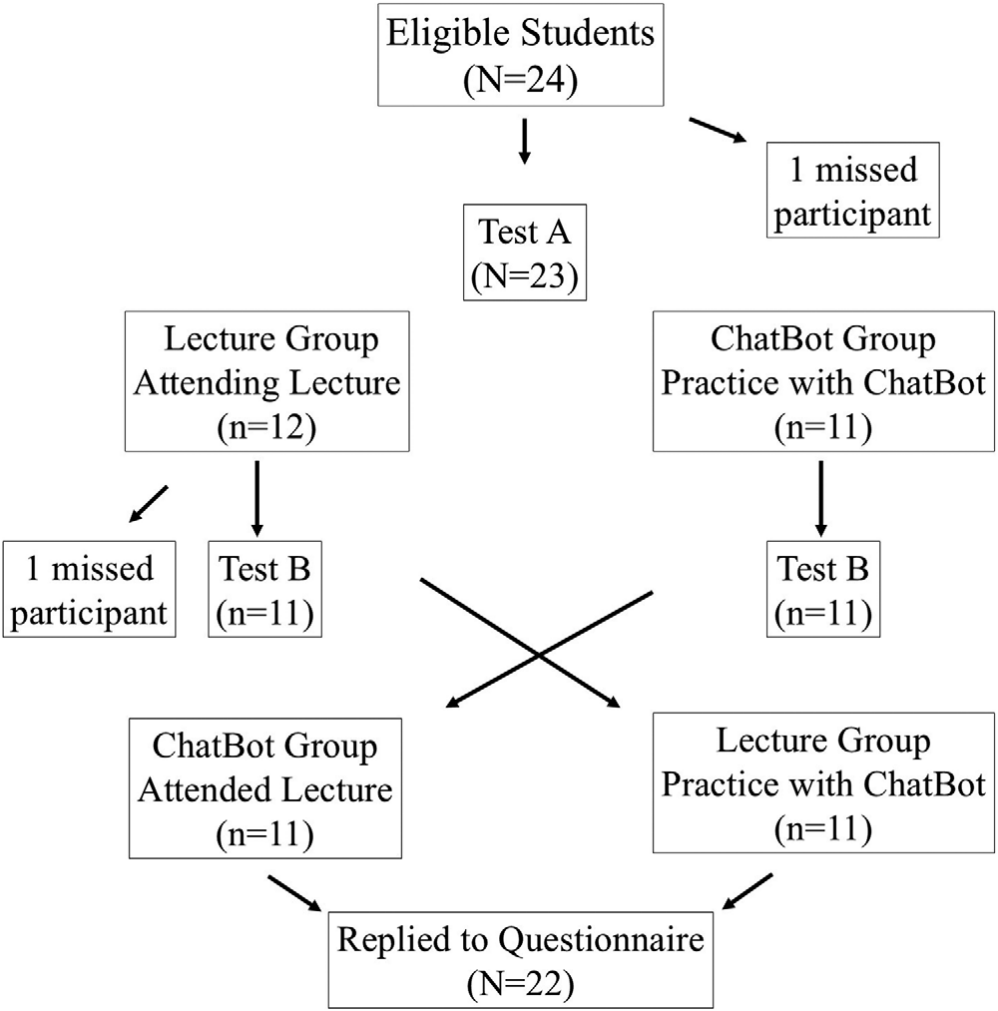
Flowchart of the experiment.

During the whole experiment the students of one group had no contact with the students of the other group from the end of Test A until the end of the questionnaire. Figure 2 summarizes the flow of the experiment.

### 2.1 Statistical Analysis

The data showed a non‐normal distribution based on the Shapiro–Wilk test. The Mann–Whitney test was used for the comparison of the grades between the two groups. The Wilcoxon test was used for the comparison of Test A and Test B for each group.

For the comparison of questionnaires, the Wilcoxon test was used. The chi‐square test was used to assess differences in the number of correct answers in the two different groups. The SPSS Statistics (Statistical Package for the Social Sciences), version 26.0 was used for statistical analysis. The statistical significance was set at *p* < 0.05.

## 3 Results

Of 24 students in the class, one missed the class on the day of the research and one failed to respond appropriately to all the questions. Overall, 22 participants were considered, 11 in the Lecture group and 11 in the Chatbot group.

The overall median grade of Test A was 6 [range 3–8]; the median grade of Test B was 8.50 [range 6–10]. The combined performance of the students increased from Test A to Test B (*p* < 0.05).

**FIGURE 3:**
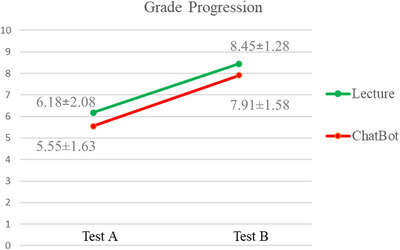
Average (SD) grades of the initial test (Test A) and final test (Test B).

The median grade on Test A of the Lecture Group was 7 [range 3–8] and the median grade of the Chatbot group was 6 [range 3–8] (*p* > 0.05). Both groups enhanced their performance after the activities without a statistically significant difference between them, Lecture group median 9 [range 6–10] and Chatbot group median 7 [range 6–10] (*p* > 0.05) (Figure 3).

There was no difference between the number of correct answers for each different question in the two different groups (*p* > 0.05).

Regarding the questionnaire, the student's perception was that the chatbot was more fun (*p* = 0.02) and simplifies the learning process (*p* = 0.015), meanwhile, the students perceived the lecture as better for understanding the content (*p* = 0.037) (Table 3).

**TABLE 3:** Comparison of the responses of the questionnaire Lecture (B) and Chatbot (A).

Questions	Strongly disagree		Disagree		Neutral		Agree		Strongly agree		*p*‐value
	Freq.	Perc.	Freq.	Perc.	Freq.	Perc.	Freq.	Perc.	Freq.	Perc.	
A1	0	0.00%	2	9.10%	3	13.60%	5	22.70%	12	54.50%	0.856
B1	0	0.00%	1	4.50%	3	13.60%	7	31.80%	11	50.00%	
A2	1	4.50%	0	0.00%	2	9.10%	3	13.60%	16	72.70%	0.078
B2	0	0.00%	2	9.10%	6	27.30%	4	18.20%	10	45.50%	
A3	0	0.00%	1	4.50%	1	4.50%	2	9.10%	18	81.80%	0.002*
B3	2	9.10%	0	0.00%	6	27.30%	8	36.40%	6	27.30%	
A4	0	0.00%	2	9.10%	4	18.20%	6	27.30%	10	45.50%	0.037*
B4	0	0.00%	0	0.00%	3	13.60%	2	9.10%	17	77.30%	
A5	1	4.50%	1	4.50%	3	13.60%	9	40.90%	8	36.40%	0.266
B5	0	0.00%	2	9.10%	1	4.50%	7	31.80%	12	54.50%	
A6	0	0.00%	0	0.00%	0	0.00%	3	13.60%	19	86.40%	0.015*
B6	1	4.50%	0	0.00%	3	13.60%	7	31.80%	11	50.00%	
A7	0	0.00%	0	0.00%	4	18.20%	5	22.70%	13	59.10%	0.560
B7	0	0.00%	2	9.10%	0	0.00%	5	22.70%	15	68.20%	
A8	1	4.50%	1	4.50%	2	9.10%	1	4.50%	17	77.30%	0.225
B8	1	4.50%	1	4.50%	5	22.70%	4	18.20%	11	50.00%	
A9	0	0.00%	0	0.00%	3	13.60%	7	31.80%	12	54.50%	0.813
B9	0	0.00%	0	0.00%	3	13.60%	6	27.30%	13	59.10%	
A10	0	0.00%	2	9.10%	1	4.50%	6	27.30%	13	59.10%	0.967
B10	0	0.00%	1	4.50%	1	4.50%	8	36.40%	12	54.50%	
A11	1	4.50%	1	4.50%	1	4.50%	9	40.90%	10	45.50%	0.552
B11	1	4.50%	0	0.00%	0	0.00%	10	45.50%	11	50.00%	
A12	1	4.50%	2	9.10%	6	27.30%	7	31.80%	6	27.30%	0.515
B12	1	4.50%	1	4.50%	6	27.30%	6	27.30%	8	36.40%	

Abbreviations: Freq, frequency; Perc, percentage.

* indicates statistically significant differences.

One additional question was applied to the chatbot questionnaire asking if they thought that chatbot was easy to use. Twenty‐one out of 22 students strongly agreed with this statement and one student agreed with the statement.

**FIGURE 4:**
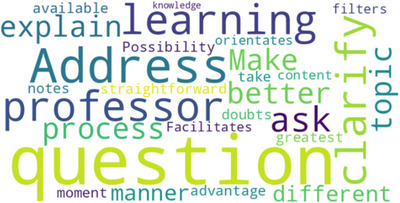
Word cloud generated by the responses of the students about the advantages of the lecture.

**FIGURE 5:**
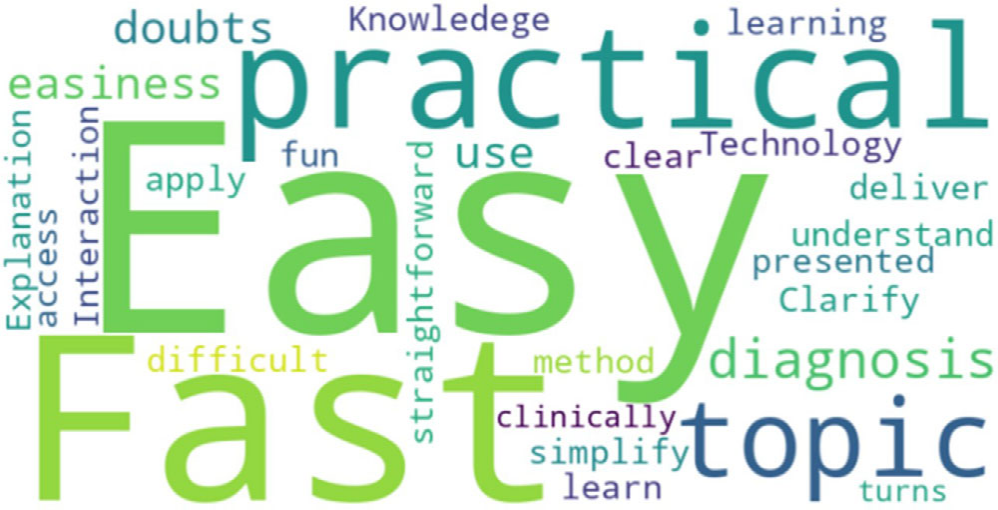
Word cloud generated by the responses of the students about the advantages of the chatbot.

All participants of the Lecture group replied to the open‐ended questions, meanwhile only five participants of the Chatbot group replied to the questions. Tables 4 and 5 depict the responses collected. Figures 4 and 5 represent the word cloud generated by the responses of the lecture and chatbot respectively.

**TABLE 4:** Response of the Lecture group about the drawbacks and advantages of both methods.

Group Lecture		
	Major drawbacks using the Chatbot	Greatest advantage of the Chatbot
1	None	Fast learning
2	I cannot report	Easy to use
3	None	Technology and easiness
4	Retaining the content	Clarify doubts on diagnosis
5	Limited answers	Easy to use
6	Retain the knowledge and obtain further details on the topic	—
7	Using my knowledge	Easy to diagnosis
8	None	Diagnosis
9	As the answers were yes or no, and we also attended the lecture, to topic got mixed up	Easy to apply clinically
10	None now, maybe internet access might be an issue	Topic turns easy, fast
11	None	Fast and practical

	Major drawbacks of the Lecture	Greatest advantage of the Lecture
1	Focus	I could ask questions and have the professor explain in a different manner
2	Keep focus for a long period of time	I could clarify my questions
3	Focus, concentration	Address the questions
4	Make questions in the presence of colleagues, follow the content when it is delivered fast	Explain the topics, the learning process is straightforward
5	I felt no difficulties	Possibility to address questions
6	Concentration	Make questions
7	Focus during the whole class	Make questions and take notes
8	Focus	Address the questions
9	Keep myself focused on very complex topics	Better learning of the topic
10	Keep myself concentrated during the class without getting distracted throughout	Facilitates the learning process, filters the content, and orientates the knowledge
11	It is boring	The professor, because he can address the questions

**TABLE 5:** Response of the Lecture group about drawbacks and advantages of both methods.

Group ChatBot		
	Major drawbacks of the ChatBot	Greatest advantage of the ChatBot
1	Accessing was not so easy, some students had issues downloading the app	Easy to understand, practical, simplify difficult topics
2	—	—
3	None	Knowledge deliver fast and practical on the topic presented
4	None	Explanation clear and straightforward
5	Getting lost in topics I had already responded	Practical and fun method to learn
6	—	Fast access to the topic with more doubts
7	—	Interaction, easiness
8	—	—
9	—	—
10	—	—
11	Sometimes it is a little difficult to get the exact answer for all cases, I think it could go deeper in the symptoms	The greatest advantage is that it is easy to use, and it is very straightforward, so it can be used quickly in the clinic

	Major drawbacks of the Lecture	Greatest advantage of the Lecture
1	Concentration, visual tiredness	Having the professor available to clarify my questions
2		
3		
4	None	It is better to clarify my doubts
5	None	
6		
7		
8		
9		
10		
11	My greatest challenge was to keep myself focused 100% of the time without distraction, it is monotonous	The greatest advantage is that I can ask any question at any moment

## 4 Discussion

This study aimed to assess the impact of a chatbot in the learning of endodontic diagnosis by 2nd‐year undergraduate dental students. Except for a 50‐min lecture delivered in the 1st year, this is the first contact of the students with the topic of diagnosis in endodontics. Therefore, a simplified version of a test used previously was applied [11]. The 10‐question test used clinical cases and straightforward questions to assess their knowledge. The focus of the research was to investigate whether the chatbot could enhance their learning on the topic. When compared to the Lecture group, the students of the Chatbot group could similarly enhance their grades when the second test was applied. Therefore, null Hypothesis 1 failed to be rejected.

Artificial intelligence has been the topic of different recent research, and despite its development, its use in healthcare has been criticized [14]. The validity of the answers provided by GPT‐3.5, Google Bard, and Bing presented several limitations in the field of endodontics [8]. The open source of the data provides almost unlimited information; on the other hand, the possible misinformation provided by open AI chatbots is a point that dental associations should be aware of [8]. The model proposed by the present study is a traditional chatbot, with all the questions and answers provided by the developer using a limited source of information. This prevents the possibility of misinformation, assuring that all the data provided is reliable. The students are guided through a combination of clinical questions and responses to sensibility tests, palpation, and percussion to reach a possible diagnosis. It is worth noting that one of the criticisms by the students who used the chatbot is that the information is limited in opposition to the lecture when the professor is available to provide further information and clarify any questions.

The perception of the students in a specific learning methodology can clearly impact their learning process [15]. In the present study, the perception of the students was different for the lecture and the chatbot, therefore the null Hypothesis 2 was rejected. Overall, the students found that the chatbot was better at simplifying complex topics and more fun than the lecture. On the other hand, the lecture was better for understanding the topic.

The lecture style has a key role in the learning process, in the present study the same faculty delivered the content for both groups. A previous study pointed out that when lectures are shorter than 1 h 5 min and with professor–student interaction they work better to maintain focus [15]. Moreover, a recent study pointed out that undergraduate students still prefer face‐to‐face learning [15, 16]. Indeed, the lecture adopted in the present study is within the preferred fashion and resulted in a good perception of understanding by the students.

In the field of STEM (Science, Technology, Engineering, and Mathematics), the extensive use of lectures has been questioned due to the smaller efficacy when compared to active methods [17]. In a recent study, designed with similarities with the present study, Zhou et al. compared the use of the WeChat‐based flipped classroom with the traditional lecture [10]. The findings of that study emphasize the importance of replacing lectures for more active methods of teaching. Their students performed better when the WeChat was used in contrast with the students that attended lectures. The flipped classrooms, however, can be time‐consuming, demanding more faculty to deliver the content. In the present study the same maximum time was established for the lecture or the chatbot. However, when the chatbot was used all students felt confident to respond to the test before the 50 min‐deadline. Even though the time to complete the task was not the aim of the study, this is in accordance with the replies of the students that chatbot is simpler to deliver the topic. It is our understanding that chatbot can be a useful tool to deliver the initial fundamentals of endodontic diagnosis and further discussion in small groups with faculties could be used to a deeper understanding of the topic [18].

After each activity, the students were asked to respond to a questionnaire and write about the greatest drawbacks and the greatest advantages of each method. Lectures are known to be boring, especially when a PowerPoint presentation is used; moreover, the mere expectation of boredom leads to lower concentration [19, 20]. Indeed, most of the students reported that it was difficult to maintain focus during the PowerPoint‐delivered lecture. On the other hand, the greatest advantage of the lecture, mentioned by 10 out of 14 students, was the fact that the faculty could take questions and go deeper into the topic. This points out that an interactive lecture might mitigate its intrinsic monotonous aspect. Interestingly, only five students of the Chatbot Group replied to the open‐ended questions. These students had the lecture as a second activity and then filled out the questionnaire, we can hypothesize that the tiring characteristic of the lecture discouraged the students from writing the answers.

Regarding the chatbot, almost half of the participants responded that the chatbot had no drawbacks and two participants demonstrated concerns with technical issues. As the chatbot was a self‐guided tool, few participants reported that the chatbot appeared to be confusing, the topics got mixed up, and the topic was not fully complete. These comments draw attention to the improvements necessary to the content delivered by chatbot, enhancing content, and assuring that the students covered all the topics of the subject. According to the students, the greatest advantage of the chatbot was the easiness to use. The participants of the study fall within the definition of digital natives proposed by Prensky [21]. A different pedagogy urges for a different generation and a great challenge for educators that are not digital natives is to cope with technology to enhance education [22, 23].

In the present study the whole activity was developed in a single morning. Moreover, only the theoretical aspect of the topic was assessed. This is a different approach than the proposed by Zhou et al. [10]. In that study several tools were included such as small group discussions in a flipped classroom followed by practical activities resulting in a better performance when compared to the lecture‐based group. In the present study only one tool, the chatbot, was assessed. The simpler methodology allowed the measurement of a single tool, preventing the influence of confounding factors. The findings of the present study point that the chatbot was similar to the lecture for the initial comprehension of the topic. These results cannot be extrapolated for the retention of knowledge or to the clinical abilities achieved, which merits further studies for evaluation.

The limitations of the present study are clear, endodontic diagnosis is a complex process with several variables. The activities and tests applied limited to assessing the competency achieved on this topic. In the 2nd year the students are still performing preclinical endodontic activities, they have overall little clinical activities, and the knowledge is still theoretical. The curriculum of our institution is designed in a spiral fashion, offering further lectures, active methodologies, and clinical practice [24]. To achieve the minimal knowledge that is expected by a general practitioner the topic will be revisited and deepened in content [25].

Within the limitations of the present study, it can be concluded that the chatbot was as effective as an interactive lecture in delivering the basic content of pulpal and periapical diagnosis. The students' perception was that the chatbot was simpler and more fun than the lecture; however, the interactive lecture is a better tool to fully understand the topic. The professor is irreplaceable when discussing the content.

## Supporting information




Supporting Information
