# Supplementary material for: Chatbot Versus Lecture in the Teaching of Endodontic Diagnosis for Undergraduate Students—A Pilot Study
Source: J Dent Educ. 2025 May 19;90(1):128–35. doi: 10.1002/jdd.13940 (PMC12800726; doi:10.1002/jdd.13940)
Supplement: Supplementary file 1 — Supporting Information [file JDD-90-128-s001.docx]

Manychat workflow


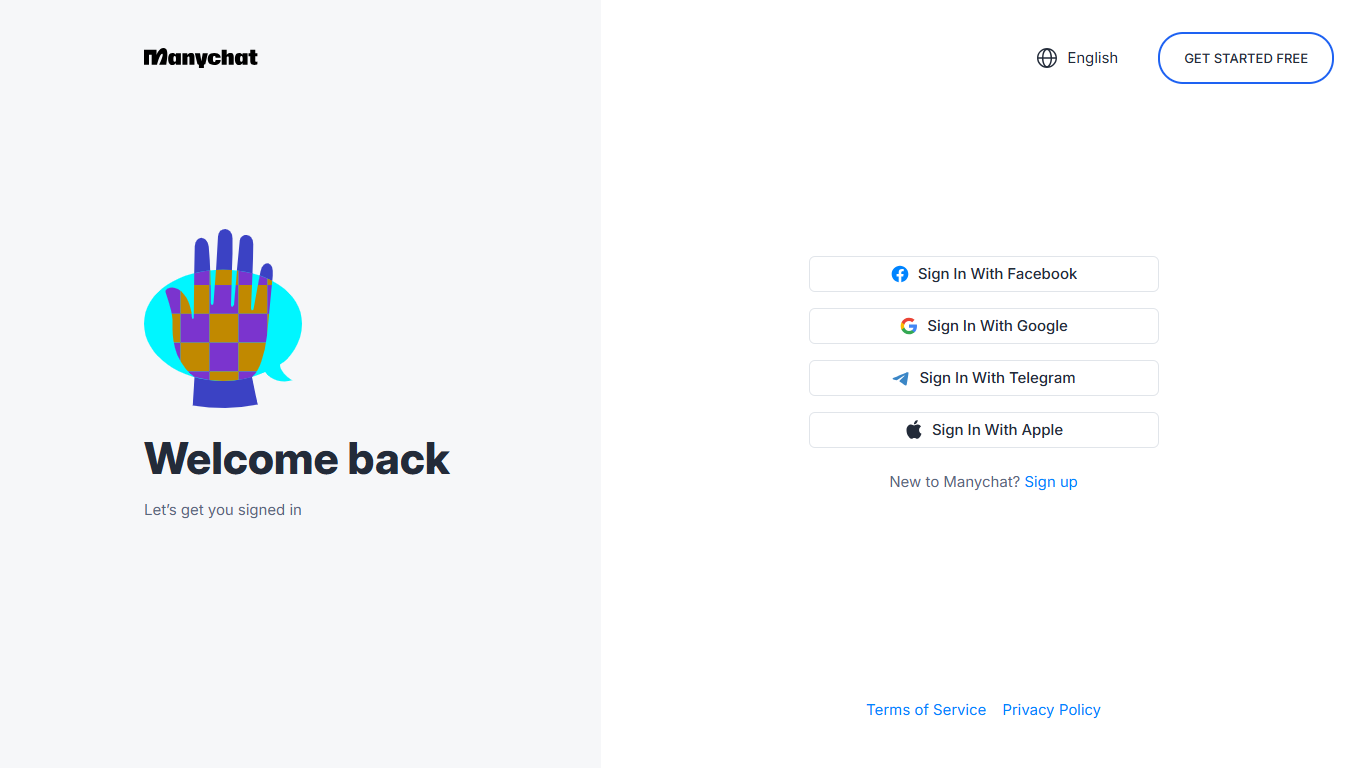


1. Go to app.manychat.com and sign in with Facebook, Google, Telegram or Apple.


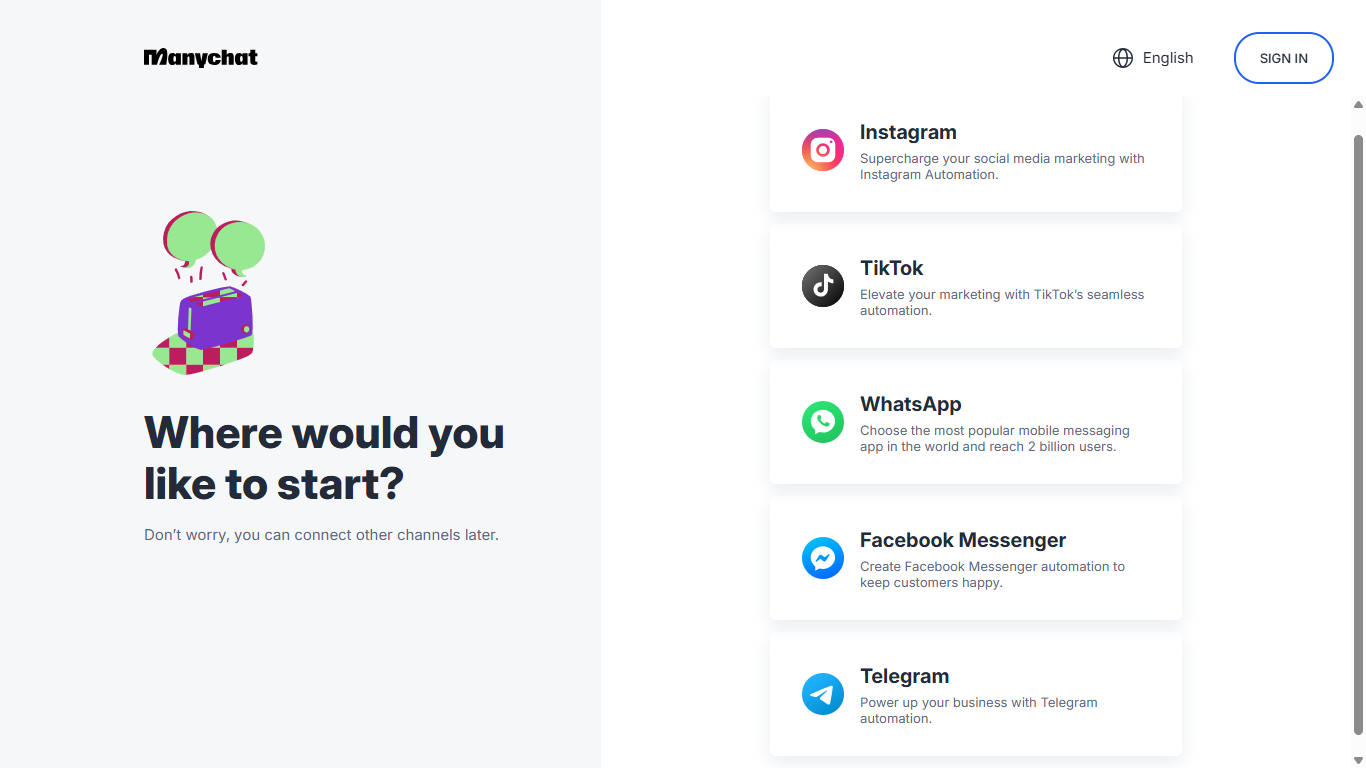


1. Choose one of the social media platforms you want to run the chatbot

**
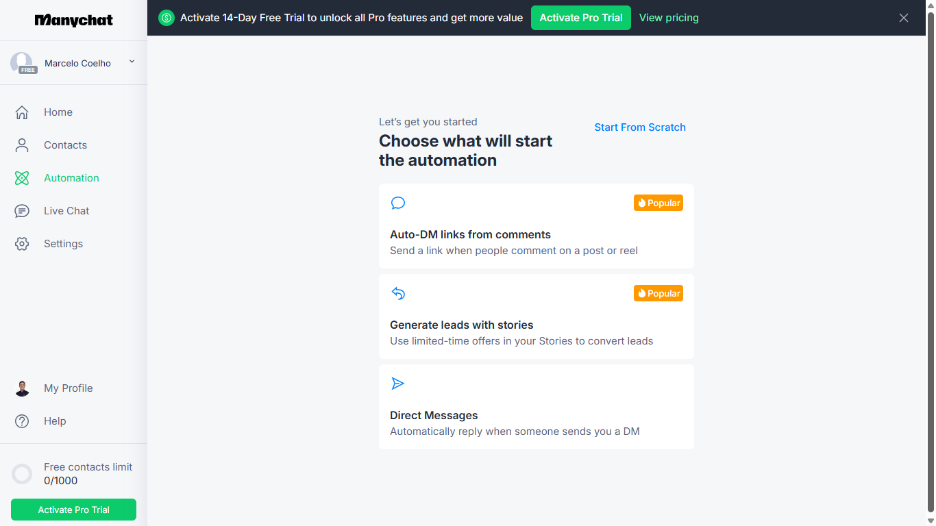
**

1. On the top left click “automation” and then “new automation” on the top right of the screen

**
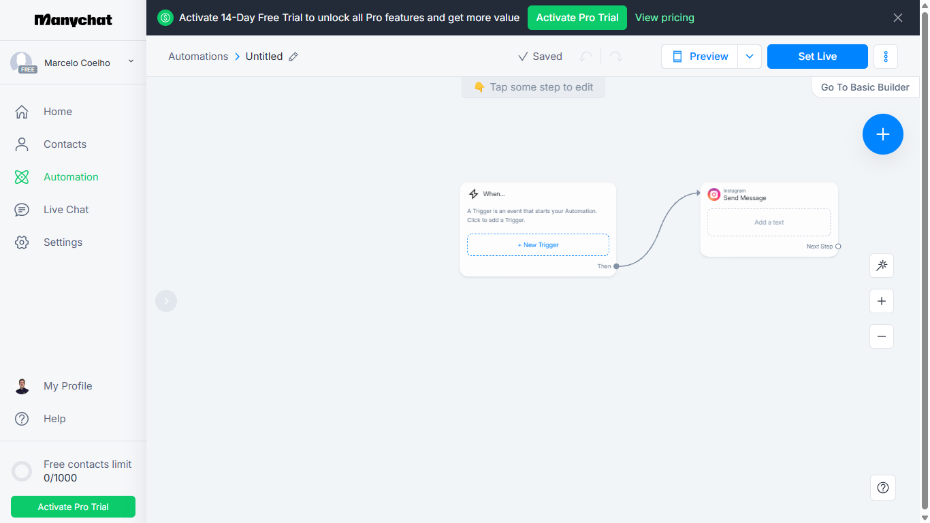
**

1. To start a new automation a trigger is created which will be responsible for running in the Telegram application
2. New buttons and texts can be added following the dialogue of choice
3. After the automation is created, click on the right top bottom and the chatbot is ready to run in the platform of choice.

Samples of the chatbot

The chatbot was developed using the Manychat (ManyChat, San Francisco, USA) program to be used in the Telegram Application (Telegram Messenger Inc., Dubai, United Arab Emirates), it can be viewed searching for the “@EndoMandicBot” profile.

The content can be translated, adapted, reviewed and applied to different realities and more complex cases. Moreover, the chatbot can run in different message applications such as WhatsApp, Instagram and Messenger. Below we present samples of interactions between the chatbot, and students related to the pulp conditions.


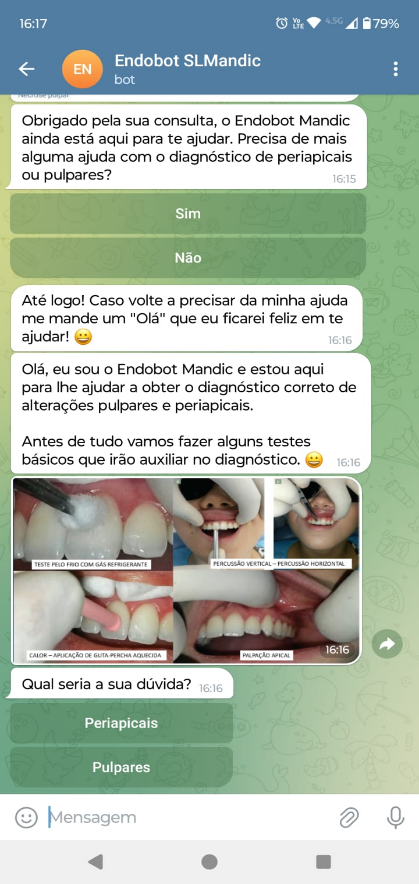

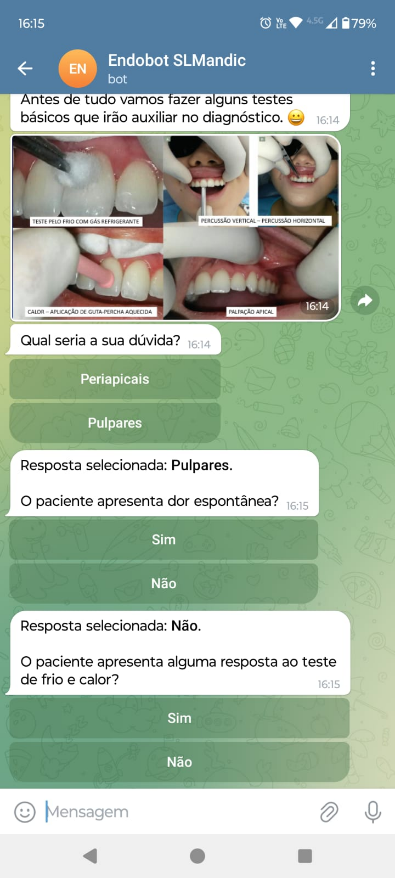

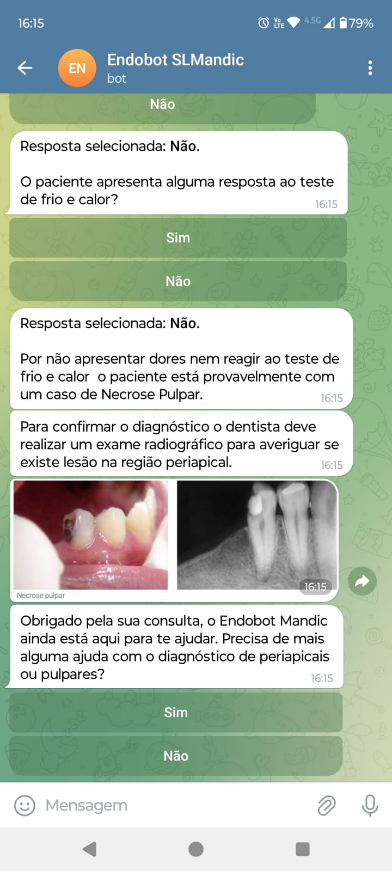


From left to right:

A) initial page greeting the student and saying that the chatbot will help with the proper diagnosis of pulpal and periapical diseases. The picture depicts a brief review of the cold/warm tests as well as horizontal/vertical percussion tests and palpation. This content was delivered to the students during their 1^st^ year.

B) By choosing “pulp” the student is lead to the second screen, the student informed that the patient has no spontaneous pain; then, the student informed that the patient is unresponsive to cold and warm tests.

C) the last screen indicates that the lack of response to sensitivity tests is likely linked to pulp necrosis and suggests a radiograph to search for radiographic features of apical periodontitis.


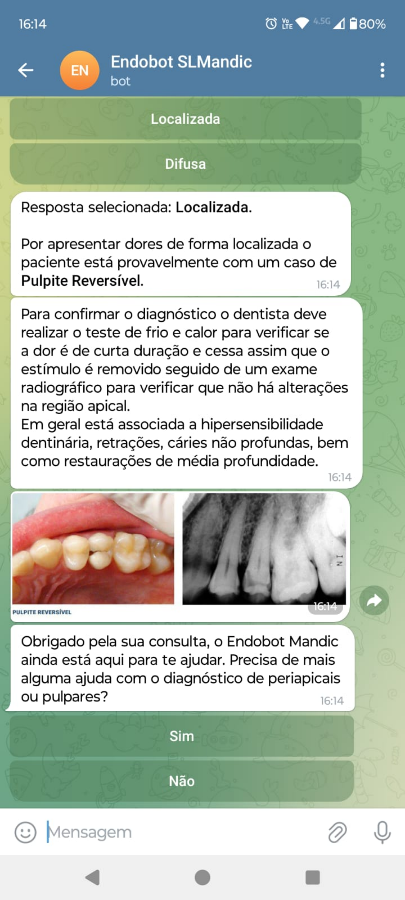

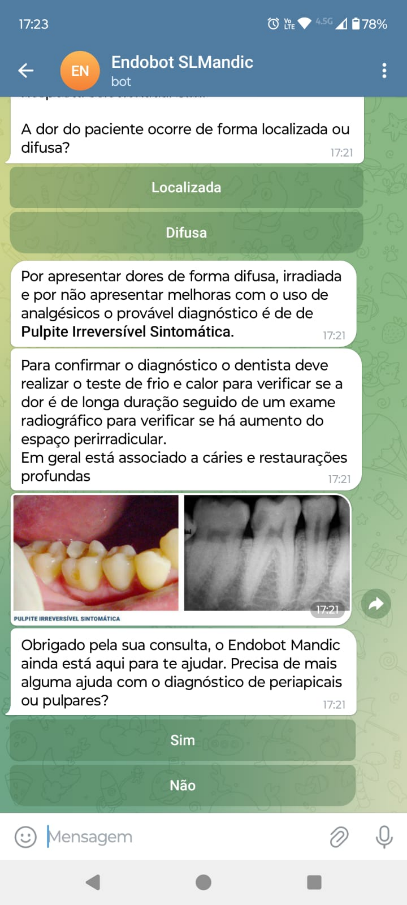

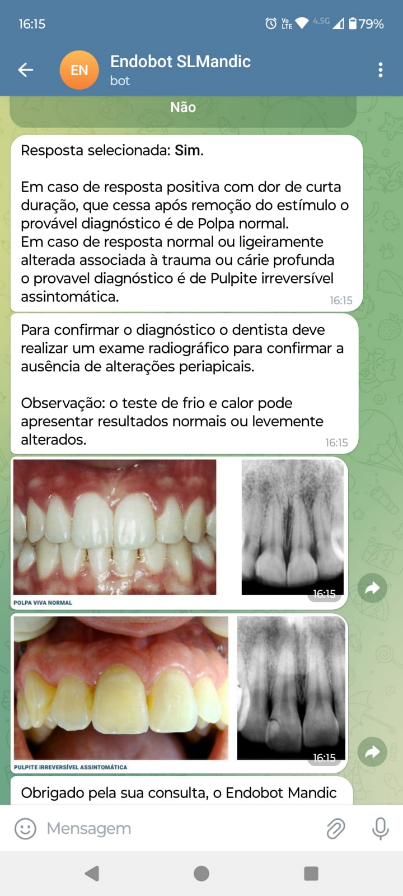


The pictures above show examples of reversible pulpitis, symptomatic irreversible pulpitis, normal pulp or asymptomatic irreversible pulpitis.
